# Health-related quality of life among patients with knee osteoarthritis in Guangzhou, China: a multicenter cross-sectional study

**DOI:** 10.1186/s12955-023-02133-x

**Published:** 2023-05-27

**Authors:** Jinghui Chang, Yuxin Yuan, Manru Fu, Dong Wang

**Affiliations:** 1 School of Health Management, Southern Medical University, Guangzhou, 510515 China; 2 Department of Biostatistics, State Key Laboratory of Organ Failure Research, Ministry of Education, and Guangdong Provincial Key Laboratory of Tropical Disease Research, School of Public Health, Southern Medical University, Guangzhou, 510515 China; 3 The Third Affiliated Hospital, Southern Medical University, Guangzhou, 510282 China

**Keywords:** Health-related quality of life, Knee osteoarthritis, Knee function, EQ-5D-5L, Guangzhou

## Abstract

**Purposes:**

To investigate health-related quality of life (HRQoL) of patients with knee osteoarthritis (KOA) in Guangzhou, China, and examine its association with selected sociodemographic characteristics as well as knee function.

**Methods:**

This multicenter cross-sectional study included 519 patients with KOA in Guangzhou from April 1 to December 30, 2019. Data on sociodemographic characteristics were obtained using the General Information Questionnaire. The disability was measured using the KOOS-PS, resting pain using the Pain-VAS, and HRQoL using the EQ-5D-5L. The association of selected sociodemographic factors, KOOS-PS and Pain-VAS scores with HRQoL (EQ-5D-5L utility and EQ-VAS scores) were analyzed using linear regression analyses.

**Results:**

The median (interquartile range [IQR]) of EQ-5D-5L utility and EQ-VAS scores were 0.744 (0.571–0.841) and 70 (60–80) respectively, lower than the average HRQoL in the general population. Only 3.661% of KOA patients reported no problems in all EQ-5D-5L dimensions, with Pain/Discomfort being the most frequently affected dimension (78.805%). The correlation analysis showed that the KOOS-PS score, Pain-VAS score and HRQoL were moderately or strongly correlated. Patients with cardiovascular disease, no daily exercise, and high KOOS-PS or Pain-VAS scores had lower EQ-5D-5L utility scores; and patients with body mass index (BMI) > 28 ,high KOOS-PS or Pain-VAS scores had lower EQ-VAS scores.

**Conclusions:**

Patients with KOA had relatively low HRQoL. Various sociodemographic characteristics as well as knee function were associated with HRQoL in regression analyses. Providing social support and improving their knee function through methods such as total knee arthroplasty might be crucial to improve their HRQoL.

## Introduction

Osteoarthritis (OA) is a common and disabling chronic musculoskeletal disease worldwide, imposing heavy social and economic burdens on patients and healthcare systems in numerous countries [1]. So far, nearly 400 million people around the world have lived with OA [1–3]. Knee osteoarthritis (KOA) is the most common clinical OA (86.8%), characterized by degeneration and destruction of articular cartilage and bone hyperplasia [2–4]. The Knee Injury and Osteoarthritis Outcome Score (KOOS-PS) [5] and the Visual Analogue Pain Scale (Pain-VAS) [6] are the tools commonly used to measure the knee joint function of patients with KOA. KOA patients often suffer from decreased self-care ability and even final disability due to stiffness, joint pain and limited mobility. The total prevalence of KOA among the population over 40 years old in China is 17.0%, among which the prevalence rate of men is 12.3%, and those of women is 22.2%, both of which are higher than the world average [7]. Moreover, the prevalence of KOA is showing a younger trend. The age with the highest prevalence rate has been advanced from the elderly population over 60 years old to middle-aged people aged 45–56 [8]. It is projected that the prevalence rate of people over 45 years old will rise from 13.8% to 15.7% by 2032 [9]. As a degenerative disease, KOA seriously affects the health-related quality of life (HRQoL) of middle-aged and elderly people [10, 11], and causes a certain social and economic burden [3, 12].

The HRQoL is a subjective assessment of the physical, psychological and social dimensions of health [13]. To date, several different tools have been used to measure HRQoL, including the EuroQol 5-dimension (EQ-5D) [14], the Short-Form 6-dimension (SF-6D) [15], and the Health Utilities Index (HUI) [16]. These instruments convert the subject’s self-classified information into a single utility value, enabling the predictions of HRQoL and comparisons between different populations [17]. According to the National Institute for Health and Care Excellence (NICE) guidelines [17], the EQ-5D is the most widely used tool for estimating HRQoL. The original description system of the EQ-5D has five dimensions (Mobility, Self-care, Usual activities, Pain/Discomfort and Anxiety/Depression) with three response levels for each dimension (EQ-5D-3L) [18]. To reduce the potential ceiling effect and increase sensitivity for detecting differences in HRQoL, the EuroQol Group introduced the 5-level version of the EQ-5D (EQ-5D-5L) in 2009 [19]. Existing evidence suggests that the 5-level version does improve measurement properties, including ceiling effects, feasibility and sensitivity, thus may be more suitable for measuring population-level HRQoL [20–24]. The EQ-5D-5L also includes a visual analogue scale (EQ-VAS), which records the patient's self-rated health status [25]. Today, the value sets of the EQ-5D-5L are available in more than 10 jurisdictions, including England [26], Germany [27], Spain [23], Netherlands [28], Poland [29], Canada [30], Indonesia [31], Uruguay [32], Japan [33], Korea [34], Australia [18], China [35, 36] and Hong Kong (HK) [17, 37].

Despite the potentially large impact of KOA on people's health, so far relatively few studies have assessed the impact on HRQoL and its determinants [38–41]. Previous studies on KOA patients have mostly focused on the costs and benefits of surgery [42], the safety and effectiveness of postoperative rehabilitation [43], the clinical effectiveness of surgery [44, 45], or the joint function status and influencing factors of patients after treatment [7]. To the best of our knowledge, there are only a few studies investigating the HRQoL of KOA patients and its influencing factors in China, although some studies on HRQoL had been conducted in other populations (e.g., Spain [40]). Therefore, we conducted this multicenter cross-sectional study in Guangzhou, the largest city in southern China. The objectives of this study were to: (1) describe the HRQoL (i.e., EQ-5D-5L utility and EQ-VAS scores) among KOA patients in Guangzhou, using the Chinese scoring algorithm; and (2) analyze the impacts of selected sociodemographic characteristics as well as knee function (i.e., KOOS-PS and Pain-VAS scores) on HRQoL.

## Methods

### Study design and participants

This cross-sectional study included patients before total knee arthritis (TKA) at one of four tertiary hospitals in four districts of Guangzhou, China, from April 1 to December 30, 2019. We used the following inclusion criteria to filter these patients: (1) diagnosed as KOA according to the 2018 clinical guidelines for the diagnosis of osteoarthritis in China [46] and (2) having a permanent residence in Guangzhou, or a long-term resident (more than 20 years). Patients with severe comorbidities (e.g., severe Parkinson's disease, post-stroke state, malignant tumors and end-stage renal disease), those with mental illness or slurred speech, and those who were unable to care for themselves were excluded. Finally, according to the inclusion criteria, 519 patients with KOA were included in this paper. Questionnaires were distributed to these patients through one-on-one interviews by researchers and medical students, with the complete rate being 100%.

### General information questionnaire

Sociodemographic characteristics of patients were collected using the General Information Questionnaire developed by the International Consortium for Health Outcomes Measurement (ICHOM) for KOA. The survey content included sex, age, height, weight, marital status, smoking status, alcohol consumption, monthly family income, medical insurance, frequency of physical activity, history of knee surgery, and history of KOA. Body mass index (BMI, in kg/m^2^) was calculated using height and weight for each patient. According to the guidelines of the Working Groups on Obesity in China [47], BMI was divided into the following four categories: underweight (<18.5), normal weight (18.5-23.9), overweight (24.0-27.9), and obese (≥ 28).

### Knee Injury and Osteoarthritis Outcome Score (KOOS-PS)

The Knee Injury and Osteoarthritis Outcome Score (KOOS-PS), a simplified version of the original KOOS, is a measure of impairment, handicap and disability after knee injury [5]. It includes seven dimensions, each with a difficulty rating from 0 to 4 (0, none; 1, mild; 2, moderate; 3, very; and 4, extreme). The raw total score ranges from 0 to 28, with higher scores indicating poorer joint function, in a direction consistent with pain measures developed at the same time, such as the Visual Analogue Pain Scale (Pain-VAS) score [6]. The KOOS-PS standard score is converted from the scoring formula specified in the scale, ranging from 0 to 100 [48]. The Cronbach's *alpha coefficient* of the KOOS-PS score in this study was 0.85, indicating that the internal consistency of this scale is good.

### Visual Analogue Pain Scale (Pain-VAS)

The Visual Analogue Pain Scale (Pain-VAS) score is mostly used for the subjective feelings of the respondents on pain. It uses a 10-point scale, with higher scores indicating greater pain intensity, such as 0 for no pain and 10 for severe pain [5].

### European Quality of Life Five Dimension Five Level Scale (EQ-5D-5L)

The European Quality of Life Five-Dimensional Five-Level (EQ-5D-5L) scale is a standardized tool developed by the EuroQol Group to measure HRQoL [4]. Because of its simple operation, easy-to-understand content, and good reliability and validity, the EQ-5D-5L scale is widely used in the world to evaluate the health status of people with chronic diseases. The scale contains five dimensions (Mobility, Self-care, Usual activities, Pain/Discomfort and Anxiety/Depression), and each is scored from 1 to 5 (1, no problem; 2, slight problems; 3, moderate problems; 4, severe problems; and 5, extreme problems). Thus, 3125 possible health states are defined by combining the scores of each dimension, ranging from 11,111 (perfect health) to 55,555 (worst health). The EQ-5D-5L health status is converted into a single "utility" score using the Chinese scoring algorithm, the utility score range from -0.391 to 1.000, with higher scores indicating better health status [35]. The Cronbach's *alpha coefficient* of the EQ-5D-5L utility score in this study was 0.78, representing that the internal consistency of this scale is good. This EQ-5D-5L scale also includes a visual analogue scale (i.e., EQ-VAS), which records the patient's self-rated health status [25]. The EQ-VAS score range from 0 to 100, in a direction consistent with the EQ-5D-5L utility score, with higher scores representing better health status.

### Statistical analysis

Descriptive summary statistics were performed for sociodemographic characteristics, KOOS-PS score, Pain-VAS score, EQ-5D-5L dimensions (Mobility, Self-care, Usual activities, Pain/Discomfort and Anxiety/Depression), EQ-5D-5L utility score, EQ-VAS score, and the top 20 most frequent EQ-5D-5L health status [18]. Univariate analysis was used to explore differences in HRQoL (i.e., EQ-5D-5L utility and EQ-VAS scores) among sociodemographic subgroups, when data were homogeneous, we used the Student's t-test (two groups) and Fisher's classic one-way ANOVA (multiple groups); when data were heterogeneous, we used the Welch's t-test (two groups) and Welch's ANOVA (multiple groups) [49]. The homogeneity of variance was tested using Levene's test. Please see notes in Table 1 to clarify which data for homogeneous or heterogeneous. The Spearman's rank correlation coefficient was used to describe the pairwise correlation among the KOOS-PS score, Pain-VAS score, EQ-5D-5L dimensions, EQ-5D-5L utility and EQ-VAS scores. Generally speaking, the absolute value of the correlation coefficient <0.30 was considered a weak correlation, 0.30-0.50 moderate and >0.50 strong [18]. Lastly, the impacts of statistically significant sociodemographic characteristics in the univariate analyses as well as knee function (KOOS-PS and Pain-VAS scores) on HRQoL were explored using the linear regression models.*p-*value < 0.05 (two-sided) was considered statistically significant. The database was established using EpiData 3.1 software, and R software (version 4.1.2) was used for data cleaning, statistical description, and statistical analysis.

**Table 1:** Sociodemographic characteristics of KOA patients and their association with HRQoL (i.e., EQ-5D-5L utility and EQ-VAS scores)

Characteristic	$\mathrm{No}. (\mathrm{\%})$	EQ-5D-5L utility score		EQ-VAS score	
		mean ± sd	*p*	mean ± sd	*p*
Location					
Hospital A	164 (31.599)	0.713 $\pm$ 0.224	< 0.001	66.793 $\pm$ 16.789	0.434
Hospital B	96 (18.497)	0.642 $\pm$ 0.246		69.740 $\pm$ 17.137	
Hospital C	237 (45.665)	0.720 $\pm$ 0.211		68.418 $\pm$ 15.184	
Hospital D	22 (4.239)	0.356 $\pm$ 0.249		65.636 $\pm$ 9.810	
Sex					
Male	115 (22.158)	0.709 $\pm$ 0.269	0.275	70.565 $\pm$ 17.248	0.053
Female	404 (77.842)	0.682 $\pm$ 0.225		67.309 $\pm$ 15.443	
Age, years					
< 60	125 (24.085)	0.744 $\pm$ 0.182	< 0.001^‡^	69.080 $\pm$ 18.070	0.655
60–74	291 (56.069)	0.691 $\pm$ 0.240		67.526 $\pm$ 14.971	
> 74	103 (19.846)	0.611 $\pm$ 0.262		68.184 $\pm$ 15.742	
Marital status					
Unmarried	59 (11.368)	0.650 $\pm$ 0.272	0.186	67.203 $\pm$ 16.534	0.672
Married	460 (88.632)	0.693 $\pm$ 0.230		68.137 $\pm$ 15.834	
Smoking status					
No	487 (93.834)	0.691 $\pm$ 0.230	0.446^‡^	67.992 $\pm$ 15.941	0.828
Yes	32 (6.166)	0.647 $\pm$ 0.314		68.625 $\pm$ 15.523	
Alcohol consumption					
No	494 (95.183)	0.687 $\pm$ 0.235	0.844	68.113 $\pm$ 15.674	0.600
Yes	25 (4.817)	0.697 $\pm$ 0.262		66.400 $\pm$ 20.189	
Income, CNY ^+^					
< 2000	245 (48.134)	0.683 $\pm$ 0.251	0.060	67.053 $\pm$ 16.729	0.105
2000–4000	187 (36.739)	0.676 $\pm$ 0.228		68.551 $\pm$ 14.387	
> 4000	77 (15.128)	0.749 $\pm$ 0.192		71.364 $\pm$ 15.317	
Medical insurance					
No	477 (91.908)	0.684 $\pm$ 0.236	0.203	68.388 $\pm$ 15.934	0.085
Yes	42 (8.092)	0.732 $\pm$ 0.235		63.976 $\pm$ 15.120	
Duration of illness, years					
< 5	232 (44.701)	0.690 $\pm$ 0.255	0.111	69.151 $\pm$ 16.631	0.216
6–15	210 (40.462)	0.704 $\pm$ 0.211		67.690 $\pm$ 15.525	
> 15	77 (14.836)	0.638 $\pm$ 0.234		65.584 $\pm$ 14.480	
Cardiovascular disease					
No	487 (93.834)	0.700 $\pm$ 0.224	0.003^‡^	68.372 $\pm$ 15.916	0.057
Yes	32 (6.166)	0.511 $\pm$ 0.325		62.844 $\pm$ 14.975	
Total knee arthroplasty					
No	468 (90.173)	0.695 $\pm$ 0.227	0.111^‡^	68.325 $\pm$ 15.844	0.202
Yes	51 (9.827)	0.625 $\pm$ 0.299		65.333 $\pm$ 16.332	
Other joint diseases					
No	462 (89.017)	0.693 $\pm$ 0.232	0.196	68.253 $\pm$ 15.840	0.365
Yes	57 (10.983)	0.650 $\pm$ 0.261		66.228 $\pm$ 16.419	
Trauma or ligament injury					
No	423 (81.503)	0.690 $\pm$ 0.236	0.711	67.809 $\pm$ 16.173	0.504
Yes	96 (18.497)	0.680 $\pm$ 0.235		69.010 $\pm$ 14.683	
Daily exercise, minutes					
None	183 (35.260)	0.607 $\pm$ 0.281	< 0.001^‡^	67.169 $\pm$ 14.334	0.321
< 30	129 (24.855)	0.738 $\pm$ 0.183		67.178 $\pm$ 16.990	
≥ 30	207 (39.884)	0.728 $\pm$ 0.200		69.324 $\pm$ 16.496	
BMI,$\mathrm{kg}/{\mathrm{m}}^{2}$					
< 18.5	6 (1.156)	0.378 $\pm$ 0.355	0.002	58.500 $\pm$ 11.895	0.025
18.5–24	142 (27.360)	0.727 $\pm$ 0.214		70.824 $\pm$ 15.804	
24–28	211 (40.655)	0.682 $\pm$ 0.244		68.000 $\pm$ 16.560	
> 28	160 (30.829)	0.673 $\pm$ 0.230		65.950 $\pm$ 14.861	

*Abbreviations*: *EQ-5D-5L* utility score, a single index ‘utility’ score representing the EQ-5D-5L health states, *EQ-VAS score* EQ visual analogue scale; sd, Standard Deviation

^+^100 CNY equals approximately 14.9 USD (April 2019 exchange rate)

^‡^Comparisons of the EQ-5D-5L utility score distributions by age, smoking status, cardiovascular disease, total knee arthroplasty, or daily exercise were analyzed using the Welch's t-test (two groups) or the Welch's ANOVA (multiple groups), since their corresponding $p$-values of the Levene's test for homogeneity of variances are all < 0.05. All other differences among groups were analyzed with the Student's t-test (two groups) or Fisher's classic one-way ANOVA (multiple groups)

## Results

Table 1 shows the sociodemographic characteristics of KOA patients and their association with HRQoL (i.e., EQ-5D-5L utility and EQ-VAS scores). Among the 519 KOA patients, 404 (77.842%) were female, 460 (88.632%) were married, and 32 (6.166%) had cardiovascular disease. The median age was 66 years (interquartile range [IQR], 60–73 years, range, from 28–88 years), 291 (56.069%) and 103 (19.846%) were aged 60-74 and > 74, respectively. A total of 183 patients (35.260%) did not exercise daily, 129 (24.855%) exercised < 30 minutes a day, and 207 (39.884%) exercised > 30  minutes a day. In addition, 211 (40.655%) and 160 (30.829%) patients were overweight and obese, respectively. The results of the univariate analyses showed that location, age, cardiovascular disease, daily exercise and body mass index (BMI) may be associated with HRQoL(*p* < 0.05) Furthermore, the average KOOS-PS and Pain-VAS scores were 33.920 (sd = 14.739) and 3.894 sd = 2.601 respectively.

**Fig. 1:**
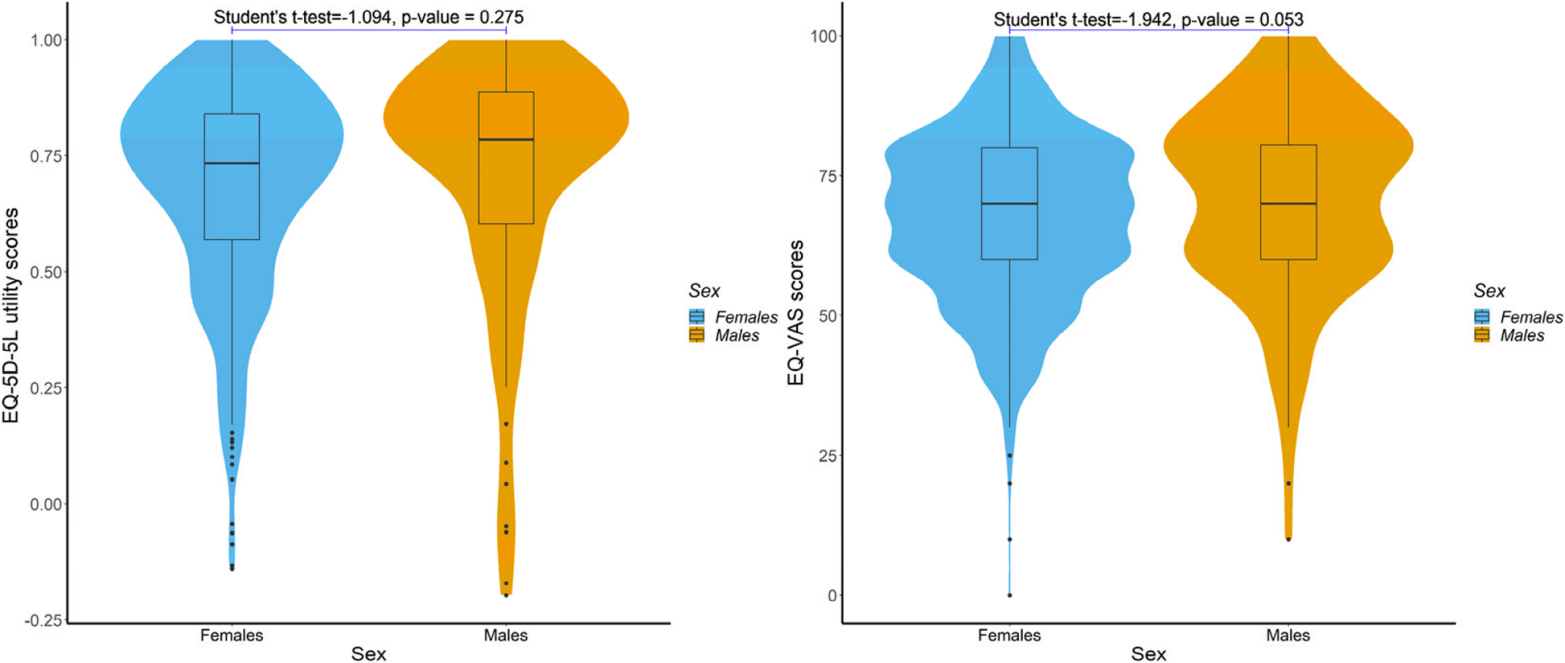
Violin plots reporting EQ-5D-5L utility scores (left) and EQ-VAS scores (right) between female and male groups, respectively. *p*-values for the differences between sexes were computed using the Student’s t-test

The violin plots in Fig. 1 report the EQ-5D-5L utility scores (left) and EQ-VAS scores (right) between female and male groups, respectively, and $p$-values for the differences between sexes were computed using the Student’s t-test. As presented in Fig. 1, the EQ-5D-5L utility scores for both females and males were heavily left-skewed, with the values concentrated between 0.6 and 0.8. The EQ-VAS scores were slightly left-skewed, and the values clustered predominantly around 60 and 80. In addition, we found no statistically significant differences in HRQoL between sexes.

Table 2 presents the frequencies (percentages%) of item responses in each EQ-5D-5L dimension (Mobility, Self-care, Usual activities, Pain/Discomfort or Anxiety/Depression) of KOA patients in Guangzhou. As shown in Table 2, the proportion of KOA patients reporting Pain/Discomfort problems was the highest (78.805%), followed by Mobility (74.952%), Usual activities (67.052%) and Anxiety/Depression (59.730%), while the proportion of reporting Self-care problem was the lowest (45.665%). The median (IQR) of EQ-5D-5L utility and EQ-VAS scores were 0.744 (0.571–0.841) and 70 (60–80), respectively, which were lower than the average HRQoL in the general population. Table 3 shows the top 20 most frequent EQ-5D-5L health states with mean EQ-5D-5L utility and EQ-VAS scores. Unlike community-based general population samples [17, 18], only 3.661% of KOA patients reported no problems in all five dimensions (health state 11,111). Additionally, 3.854% of patients rated their health status as $>90$ on the EQ-VAS. These 20 health states accounted for about half of the patients (46.243%). The top 10 health states included "11121" (6.358%), "11111" (3.661%), "21221" (3.661%), "22222" (3.661%), "21222" (2.890%), "22221" (2.890%), "21121" (2.697%), "11122" (2.505%), "11112" (2.312%) and "21122" (2.312%).

**Table 2:** Frequencies (percentages%) of item responses in each EQ-5D-5L dimension

Dimension	Item responses^a^					Item scores^b^	
	No problems	Slight	Moderate	Severe	Extreme	$\mathrm{mean}\pm \mathrm{sd}$	$\mathrm{median}\left(\mathrm{IQR}\right)$
Mobility	130 (25.048)	209 (40.270)	138 (26.590)	33 (6.358)	9 (1.734)	2.195 ± 0.944	2 (1–3)
Self-care	282 (54.335)	158 (30.443)	58 (11.175)	14 (2.697)	7 (1.349)	1.663 ± 0.879	1 (1–2)
Usual activities	171 (32.948)	235 (45.279)	82 (15.800)	26 (5.010)	5 (0.963)	1.958 ± 0.880	2 (1–2)
Pain/Discomfort	110 (21.195)	229 (44.123)	138 (26.590)	36 (6.936)	6 (1.156)	2.227 ± 0.900	2 (2–3)
Anxiety/Depression	209 (40.270)	193 (37.187)	90 (17.341)	24 (4.624)	3 (0.578)	1.881 ± 0.895	2 (1–2)

*Abbreviations*: *sd* Standard Deviation, *IQR* interquartile range

^a^Data are presented as No (%)

^b^Item scores for the five EQ-5D-5L dimensions (Mobility, Self-care, Usual activities, Pain/Discomfort and Anxiety/Depression) range from 1 to 5

**Table 3:** Top 20 most frequent EQ-5D-5L health states with mean EQ-5D-5L utility and EQ-VAS scores

Health state^a^	$\mathrm{No}.$	%	Cumulative %	Mean EQ-5D-5L utility	Mean EQ-VAS $\pm \mathrm{sd}$
11121	33	6.358	6.358	0.942	75.515 $\pm$ 12.232
11111	19	3.661	10.019	1.000	79.263 $\pm$ 16.048
21221	19	3.661	13.680	0.831	77.158 $\pm$ 17.115
22222	19	3.661	17.341	0.734	65.526 $\pm$ 17.595
21222	15	2.890	20.231	0.782	72.467 $\pm$ 10.056
22221	15	2.890	23.121	0.783	70.867 $\pm$ 11.934
21121	14	2.697	25.819	0.876	67.429 $\pm$ 14.553
11122	13	2.505	28.324	0.893	71.769 $\pm$ 13.084
11112	12	2.312	30.636	0.951	78.167 $\pm$ 10.382
21122	12	2.312	32.948	0.827	61.083 $\pm$ 21.194
22211	10	1.927	34.875	0.841	73.400 $\pm$ 12.340
22231	8	1.541	36.416	0.703	59.375 $\pm$ 17.410
32222	8	1.541	37.958	0.642	66.375 $\pm$ 14.121
11132	7	1.349	39.306	0.812	70.714 $\pm$ 12.392
21211	7	1.349	40.655	0.889	73.143 $\pm$ 17.620
21212	7	1.349	42.004	0.840	75.714 $\pm$ 13.048
11221	6	1.156	43.160	0.898	71.167 $\pm$ 10.284
33332	6	1.156	44.316	0.432	66.833 $\pm$ 15.171
11133	5	0.963	45.279	0.744	53.400 $\pm$ 22.534
11212	5	0.963	46.243	0.906	63.000 $\pm$ 15.652

*Abbreviations*: *EQ-5D-5L utility score* a single index ‘utility’ score representing the EQ-5D-5L health states, *EQ-VAS score* EQ visual analogue scale; sd, Standard Deviation

^a^Health states are defined by combining the response levels (1–5) for the five EQ-5D-5L dimensions (Mobility, Self-care, Usual activities, Pain/Discomfort and Anxiety/Depression), ranging from 11,111 (perfect health) to 55,555 (worst health)

**Fig. 2:**
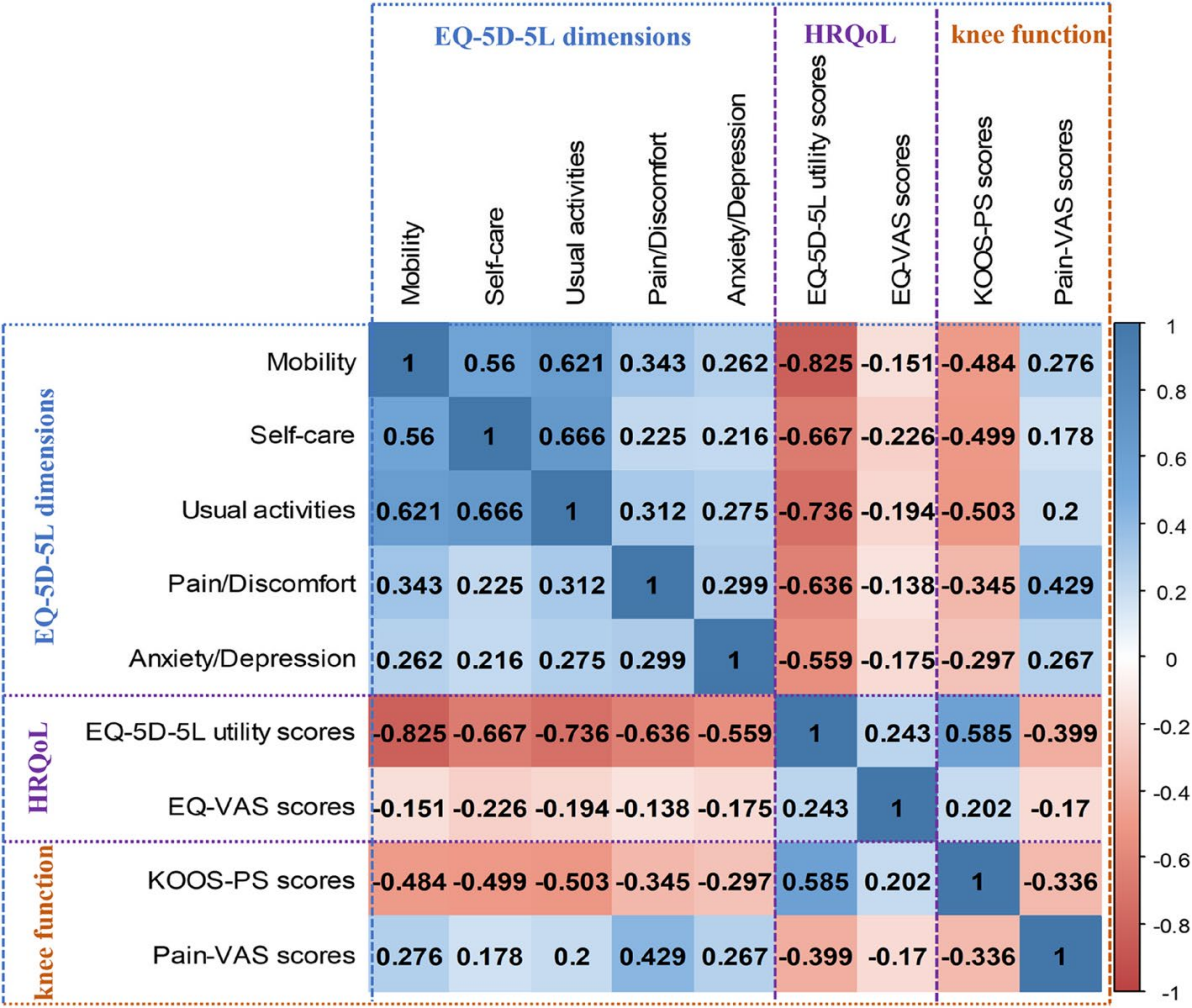
Correlations for knee function (KOOS-PS and Pain-VAS scores) and HRQoL. The Spearman's rank correlation coefficients are computed among EQ-5D-5L dimensions (Mobility, Self-care, Usual activities, Pain/Discomfort and Anxiety/Depression), EQ-5D-5L utility score, EQ-VAS score, KOOS-PS score, and Pain-VAS score. Shown in each cell is the value of the corresponding Spearman's rank correlation coefficient

Figure 2 performed the correlations for the knee function (KOOS-PS and Pain-VAS scores) and HRQoL. The Spearman's rank correlation coefficients are computed among EQ-5D-5L dimensions (Mobility, Self-care, Usual activities, Pain/Discomfort and Anxiety/Depression), EQ-5D-5L utility score, EQ-VAS score, KOOS-PS score, and Pain-VAS score. In general, there were strong positive correlations (correlation coefficients > 0.50) among Mobility, Self-care and Usual activities; strong negative correlations (correlation coefficients < - 0.50) between EQ-5D-5L utility score and EQ-5D-5L dimensions. Moreover, the EQ-VAS score was weakly correlated (the absolute value of the correlation coefficients < 0.30) with the EQ-5D-5L utility score and EQ-5D-5L dimensions. Notably, there were moderate positive correlations (correlation coefficients between 0.3 to 0.5) between the KOOS-PS score and EQ-5D-5L dimensions, a strong negative correlation (correlation coefficient = - 0.585) between KOOS-PS score and EQ-5D-5L utility score. Finally, the Pain-VAS score was moderately correlated with Pain/Discomfort, EQ-5D-5L utility, and KOOS-PS scores, where the correlation coefficients are 0.429, -0.399, and 0.336, respectively.

Next, we explored the impacts of the statistically significant sociodemographic characteristics in univariate analyses (i.e., location, age, cardiovascular disease, daily exercise and BMI), as well as knee function (KOOS-PS and Pain-VAS scores) on HRQoL using the linear regression models. Table 4 presents the results of univariate and multivariate linear regression analyses of these influencing factors associated with EQ-5D-5L utility and EQ-VAS scores, respectively. In the univariate linear regression analyses, location, age, cardiovascular disease, daily exercise, BMI, KOOS-PS and Pain-VAS scores were statistically significantly associated with EQ-5D-5L utility score ($p<0.05$); and BMI, KOOS-PS and Pain-VAS scores were statistically significantly associated with EQ-VAS score ($p<0.05$). In the multivariate linear regression analyses, the average EQ-5D-5L utility score in the hospital D was lower than that in the hospital A (regression coefficient [$\beta$]$=-0.253$; 95% confidence interval [CI], $-0.336$ to $-0.171$; $p<0.001$); the average EQ-VAS score in the hospital B was higher than that in the hospital A ($\beta =5.128$; 95% CI, $1.036$ to $9.219$; $p=0.014$). Patients with cardiovascular disease had lower EQ-5D-5L utility scores than those without ($\beta =-0.068$; 95% CI, $-0.133$ to $-0.003$; $p=0.042$). Additionally, compared with those who did not exercise daily, patients who exercised for $<30$ min daily ($\beta =0.053$; 95% CI, $0.012$ to $0.094$; $p=0.012$) or $\ge 30$ min daily ($\beta =0.046$; 95% CI, $0.008$ to $0.083$; $p=0.017$) had higher EQ-5D-5L utility scores. Patients with BMI $<18.5$ had lower EQ-5D-5L utility scores than those with BMI between 18.5 and 24, but since there were only 6 individuals (1.156%) with BMI $<18.5$, this result may not be reliable. Furthermore, the average EQ-VAS scores were lower for patients with BMI $>28$ than for those between 18.5 and 24 ($\beta =-4.768$; 95% CI, $-8.332$ to -$1.203$; $p=0.009$). Finally, we also found that the higher the KOOS-PS or Pain-VAS scores, that is, the worse the knee joint function or the more intense the subjective feeling of pain, the lower the EQ-5D-5L utility and EQ-VAS scores.

**Table 4:** Univariate and multivariate linear regression analyses of influencing factors associated with EQ-5D-5L utility and EQ-VAS scores, respectively

Variable	EQ-5D-5L utility score					EQ-VAS score			
	Univariate Models		Multivariate Model		Univariate Models			Multivariate Model	
	$\beta \left(95\% \mathrm{CI}\right)$	$p$	$\beta \left(95\% \mathrm{CI}\right)$	$p$	$\beta \left(95\% \mathrm{CI}\right)$		$p$	$\beta \left(95\% \mathrm{CI}\right)$	$p$
Location									
Hospital A	Reference		Reference		Reference			Reference	
Hospital B	-0.071 (-0.128, -0.015)	0.014	0.008 (-0.039, 0.055)	0.739	2.947 (-1.069, 6.962)		0.150	5.128 (1.036, 9.219)	0.014
Hospital C	0.006 (-0.038, 0.051)	0.782	0.008 (-0.028, 0.043)	0.660	1.625 (-1.549, 4.799)		0.315	1.575 (-1.527, 4.677)	0.319
Hospital D	-0.357 (-0.457, -0.257)	< 0.001	-0.253 (-0.336, -0.171)	< 0.001	-1.156 (-8.251, 5.938)		0.749	1.025 (-6.227, 8.277)	0.781
Age, years									
< 60	Reference		Reference		Reference			Reference	
60–74	-0.053 (-0.102, -0.005)	0.032	0.011 (-0.027, 0.049)	0.559	-1.554 (-4.899, 1.790)		0.362	0.250 (-3.056, 3.557)	0.882
> 74	-0.133 (-0.193, -0.072)	< 0.001	-0.013 (-0.061, 0.035)	0.593	-0.896 (-5.057, 3.266)		0.673	2.260 (-1.925, 6.446)	0.289
Cardiovascular disease									
No	Reference		Reference		Reference			Reference	
Yes	-0.188 (-0.271, -0.105)	< 0.001	-0.068 (-0.133, -0.003)	0.042	-5.528 (-11.214, 0.158)		0.057	-4.501 (-10.215, 1.213)	0.122
Daily exercise, minutes									
None	Reference		Reference		Reference			Reference	
< 30	0.132 (0.080, 0.183)	< 0.001	0.053 (0.012, 0.094)	0.012	0.009 (-3.581, 3.599)		0.996	-1.032 (-4.632, 2.567)	0.573
$\ge$ 30	0.122 (0.076, 0.167)	< 0.001	0.046 (0.008, 0.083)	0.017	2.154 (-1.015, 5.323)		0.182	0.593 (-2.688, 3.874)	0.723
BMI,$\mathrm{kg}/{\mathrm{m}}^{2}$									
< 18.5	-0.349 (-0.540, -0.159)	< 0.001	-0.159 (-0.306, -0.012)	0.034	-12.324 (-25.264, 0.616)		0.062	-10.066 (-22.922, 2.791)	0.125
18.5–24	Reference		Reference		Reference			Reference	
24–28	-0.045 (-0.095, 0.004)	0.073	-0.029 (-0.067, 0.009)	0.130	-2.824 (-6.194, 0.546)		0.100	-2.546 (-5.849, 0.757)	0.131
> 28	-0.054 (-0.107, -0.001)	0.045	-0.026 (-0.067, 0.015)	0.210	-4.874 (-8.454, -1.294)		0.008	-4.768 (-8.332, -1.203)	0.009
KOOS-PS, per sd^a^	-0.134 (-0.151, -0.118)	< 0.001	-0.102 (-0.119, -0.085)	< 0.001	-3.119 (-4.466, -1.772)		< 0.001	-2.479 (-3.975, -0.983)	0.001
Pain-VAS, per sd^b^	-0.089 (-0.108, -0.070)	< 0.001	-0.054 (-0.071, -0.038)	< 0.001	-2.676 (-4.031, -1.322)		< 0.001	-2.063 (-3.499, -0.626)	0.005

*Abbreviations*: *EQ-5D-5L utility score* a single index ‘utility’ score representing the EQ-5D-5L health states, *EQ-VAS score* EQ visual analogue scale; $\beta \left(95\% \mathrm{CI}\right)$, regression coefficient (95% confidence interval)

^a^KOOS-PS, sd = 14.739

^b^Pain-VAS, sd = 2.60

## Discussion

KOA is the most common clinical OA and imposes a severe burden on patients and healthcare systems. The clinical symptoms of patients with KOA are mainly knee joint swelling, pain, and mobility impairment, which severely limit the patient's mobility and self-care ability, and greatly reduce the patient's health-related quality of life (HRQoL) [50]. HRQoL is not only an indicator of physical health but also a reflection of socioeconomic and psychological status [4]. Therefore, for KOA patients, in addition to focusing on their physical discomfort and activity limitation, their HRQoL also needs to be paid attention to [51, 52]. However, relatively few studies have explored the HRQoL of KOA patients in China. This study investigated the HRQoL of KOA patients in Guangzhou and its influencing factors, including sociodemographic characteristics as well as knee function. The results of this study present that the HRQoL of patients with KOA is closely related to many factors (e.g., location, cardiovascular disease, daily exercise, BMI, KOOS-PS and Pain-VAS scores), which will be helpful for psychosocial interventions and planning health care.

Our findings demonstrated that KOA patients in Guangzhou had a relatively poor HRQoL compared with the general population, which needs attention. In this study, the median (IQR) of EQ-5D-5L utility scores was 0.744 (0.571–0.841), lower than that of the general populations in China (0.96) [36], Australia (0.91) [18], Poland (0.89) [29] and Germany (0.92) [27]. Only 3.661% of KOA patients reported no problems in all five dimensions (health state 11,111), suggesting no obvious ceiling effect on the EQ-5D-5L utility score, and it is significantly lower than the normative values estimated by EQ-5D-5L in community-based general populations such as Portugal (47%) [53], Australia (43%) [18], Germany (48%) [27], UK (48%) [20], Spain (62.4%) [23] and China (54%) [36]. Furthermore, the proportion of “no problem” responses for each EQ-5D-5L dimension were 25.048% (Mobility), 54.335% (Self-care), 32.948% (Usual activities), 21.195% (Pain/Discomfort) and 40.270% (Anxiety/Depression). The findings showed that our population experienced fewer problems in Self-care, Anxiety/Depression, and Usual activities, but greater problems in Pain/Discomfort or Mobility. The pattern is similar to other EQ-5D-5L-related studies [18, 27, 54].

The results of this study showed the correlations among EQ-5D-5L dimensions, EQ-5D-5L utility score, EQ-VAS score, KOOS-PS score, and Pain-VAS score. Specifically, we observed strong positive correlations among Mobility, Self-care and Usual activities; strong negative correlations between EQ-5D-5L utility score and EQ-5D-5L dimensions, indicating that the EQ-5D-5L has high content consistency. In addition, there were moderate positive correlations between the KOOS-PS score and EQ-5D-5L dimensions, and a strong negative correlation between the KOOS-PS score and EQ-5D-5L utility score, suggesting that the KOOS-PS score may affect the EQ-5D-5L utility score by having effects on EQ-5D-5L dimensions. Finally, the Pain-VAS score was moderately correlated with Pain/Discomfort and EQ-5D-5L utility score, indicating that the Pain-VAS score may influence the EQ-5D-5L utility score through the Pain/Discomfort dimension.

The results of multivariate linear regression analysis also showed that there were differences in the EQ-5D-5L utility and EQ-VAS scores in different hospitals, suggesting that there was a certain center specificity. Possible explanations included: the relatively small number of patients in the Hospital D (only 22), heterogeneity among the four districts, or differences in the ways the researchers asked questions. Although univariate analysis showed that age may have an effect on EQ-5D-5L. However, we did not find statistically significant association of age with HRQoL after adjusting for knee function (Table 4). We also explored the impacts of age on knee function (KOOS-PS and Pain-VAS scores) using the linear regression models, and found that age had significant negative effects on knee function. Therefore, our study did not find direct effects of age on HRQoL, although it may have effects on knee function. Also, patients with cardiovascular disease had lower EQ-5D-5L utility scores than those without. Studies have shown systematic differences in HRQoL between the general public and patients with certain health conditions, such as heart disease, and these differences cannot be explained by differences in sociodemographic characteristics [55]. Le et al. [56] conducted a systematic review and meta-analysis, and found that patients with coronary heart disease had lower HRQoL than the asymptomatic healthy participants. In addition, compared with those who did not exercise daily, patients who exercised for < 30 min daily or ≥ 30 min daily had higher EQ-5D-5L utility scores, indicating that moderate and regular exercise can both protect and improve knee function and improve EQ-5D-5L in patients with KOA [4]. Evidence also showed significant correlations between the level of physical activity and HRQoL [57, 58]. Lastly, patients with BMI > 28 had lower EQ-VAS scores than those with BMI 18.5-24 Serrano-Aguilar et.al. [59] explored the relationship between obesity and HRQoL in the general population, and found that severely obese participants had significantly lower EQ-5D-5L utility scores than normal-weight participants (0.65 vs. 0.87). Meanwhile, related studies have shown that obesity is a risk factor for KOA [60]. When the BMI value exceeds the standard, the load on the human knee joint will increase accordingly, thereby accelerating the degenerative changes of articular cartilage [61]. Therefore, KOA patients need to strengthen appropriate physical exercise, maintain a healthy diet, control the intake of oil, sugar and salt, actively control their weight, and maintain it within the normal range to reduce the pressure on the knee joint.

Furthermore, our findings found that the higher the KOOS-PS or Pain-VAS score, the lower the EQ-5D-5L utility and EQ-VAS scores, which is consistent with the previous studies [4, 41, 60]. Bilbao et.al. [41] also found that knee function and pain scores (measured by the Western Ontario and McMaster Universities Osteoarthritis Index (WOMAC) [62]) were strongly correlated with EQ-5D-5L utility scores (− 0.688 and − 0.782), and patients with higher WOMAC scores had significantly lower EQ-5D-5L utility score (*p* < 0.0001). Thus, it is crucial to improve the knee function of patients with KOA. Unicompartmental knee arthroplasty, osteotomy, arthroscopic surgery and total knee arthroplasty (TKA) can all improve the functional scores of KOA patients. Follow-up studies have shown that TKA is superior to other interventions in improving knee function and relieving knee pain in the long term [63]. Additionally, pain severely limits the patient's mobility, and can also affect the patient's psychological emotions [60]. Therefore, attention should be paid to strengthening the pain management of patients during the perioperative period. At the same time, some KOA patients with mild pain did not take timely treatment or intervention measures in the early stage of inflammation, and missed the opportunity for treatment, resulting in the aggravation of the disease [64]. In light of the aforementioned factors, pain should be effectively controlled at the early stage of the disease, combined with physical therapy intervention, to prevent acute pain from developing into uncontrollable chronic pain, which hinders early exercise. After the patient is diagnosed, doctors, nurses, patients and their family members should cooperate to standardize pain management, encourage patients to exercise properly, and improve their knee joint function [65].

### Implication

Although investigating the HRQoL of KOA patients in China is important, more studies should be conducted in this research area [38–40]. This study expands our current understanding in this area and provides evidence and suggestion for patients, families, doctors, and policymakers to improve the HRQoL of KOA patients. On the one hand, by strengthening health education, health promotion and other methods, popularizing the correct prevention methods of KOA, intervening in pain as soon as possible, and reducing the incidence of KOA. On the other hand, to improve the knee function and quality of life of KOA patients in Guangzhou, we could improve their self-health management ability, encourage them to exercise properly and maintain a normal weight, and improve their knee function through methods such as TKA. Meanwhile, more counseling, psychoeducational training or intervention, and social support programs should be offered to patients with KOA. Policymakers should promote the development of health policies for community-based mental health services [66]. Doctors, health care professionals, recovery training or intervention, and financial support are crucial for KOA patients.

### Limitations

Several limitations also need to be acknowledged. First, we only included 519 patients with advanced KOA who were going to TKA at one of four tertiary hospitals in Guangzhou, China. Those who were not planning to go to the hospital for TKA were not included in the study. These individuals may have different health indices, and this limitation may have introduced some bias in our estimates and somewhat affected the extrapolation of our study. Second, future studies should consider the impact of satisfaction subscores and patient expectations on HRQoL [67]. In addition, we only studied the HRQoL of KOA patients from a cross-sectional perspective, which does not investigate causality, and further studies are needed to plan the longitudinal design. Finally, due to the limitations of human, material, and financial resources, the research site is limited to Guangzhou, which may limit the study’s representativeness in other areas. In the future, the research site and sample size can be expanded to more comprehensively evaluate the HRQoL of KOA patients in China.

## Conclusions

In this paper, we investigated the HRQoL among patients with KOA in Guangzhou, and analyzed the influence of selected sociodemographic characteristics as well as knee function on HRQoL. In summary, the HRQoL of KOA patients in Guangzhou is relatively low, and comprehensive measures should be taken to control the occurrence and development of KOA. The HRQoL of patients with KOA is related to some sociodemographic characteristics (i.e., location, cardiovascular disease, daily exercise and BMI) as well as knee function and pain scores (i.e., KOOS-PS and Pain-VAS scores). These findings may support policymakers in maintaining the HRQoL of the Chinese population when designing community-based mental health care and health policies.

## Data Availability

Data are available from the corresponding author upon reasonable request and are strictly for research purposes.
